# Culturally Tailored Materials for Smoking Cessation and HIV Care Adherence Among Hispanic Adults: Protocol for Content Development and Formative Evaluation

**DOI:** 10.2196/81192

**Published:** 2026-04-29

**Authors:** Lorra Garey, Jacqueline J Charles, Ashley L Ruiz, Rodrigo Castillo-Avilés, Virmarie Correa-Fernández, Marshall K Cheney, Thomas P Giordano, Michael J Zvolensky

**Affiliations:** 1 HEALTH Research Institute, University of Houston, 4349 Martin Luther King Blvd, Houston, TX, 77204, United States, 1 713-743-8056; 2 Department of Psychology, University of Houston, Houston, TX, United States; 3 Department of Psychological, Health, & Learning, University of Houston, Houston, TX, United States; 4 Department of Health and Exercise Science, University of Oklahoma, Norman, OK, United States; 5 Department of Medicine, Baylor College of Medicine, Houston, TX, United States; 6 Department of Behavioral Science, The University of Texas MD Anderson Cancer Center, Houston, TX, United States

**Keywords:** Hispanic, smoking cessation, HIV, anxiety sensitivity, study protocol

## Abstract

**Background:**

Hispanic people with HIV who smoke cigarettes experience unique stressors (eg, stigma), which contribute to health disparities. Anxiety sensitivity (AS) may worsen mood management problems, which are a leading barrier to smoking cessation. Interventions targeting AS can improve HIV-specific outcomes and smoking cessation. However, no prior research has culturally tailored an AS reduction program to improve quality of life among Hispanic people with HIV who smoke. The research team previously developed a mobile health (mHealth) intervention addressing AS reduction, smoking cessation, and HIV care management for Black people with HIV who smoke. Building on this work, this study represents a formative, exploratory phase to develop culturally tailored mHealth content for Hispanic people with HIV across 3 distinct regions (Mexico, Central America, and South America), which share many similarities but differ in some cultural and linguistic respects. This work will inform the refinement of materials for these groups and the future development of an integrated mHealth app for smoking, AS, and HIV among this population (ie, VITAL).

**Objective:**

This study aimed to culturally tailor evidence-based smoking cessation content targeting AS reduction and HIV management among Hispanic adults to inform the development of the VITAL mHealth program.

**Methods:**

Intervention content was culturally adapted using a theory-informed intervention adaptation framework that integrated a cultural considerations document derived from existing literature on smoking cessation and HIV care among the Hispanic population, along with iterative consultation with a Community Research Advisory Board. This resulted in linguistically tailored content in English and Spanish. The pilot study consisted of Hispanic people with HIV who smoke (N=80), divided into 3 subgroups: Mexican/Mexican American, Central American, and South American. Participants completed self-report assessments and a semistructured interview assessing the treatment videos for content relevance, appropriateness, and ease of understanding. Interviews were conducted online in Spanish or English by trained interviewers. Interview transcripts will be coded by a multidisciplinary qualitative team using a 2-pass approach: initial coding of the interview question followed by higher-level concepts. Themes will be reviewed by another member of the team to assess trustworthiness, saturation, and triangulated with quantitative data, then analyzed by geographic subgroup.

**Results:**

Three linguistically tailored versions of the intervention materials were developed. Data collection began August 19, 2024, and finished June 26, 2025. Data cleaning is ongoing, and analyses will begin in April 2026. Content refinement and app integration are anticipated to be completed by September 2026. Upon completion of analysis, data will be used to further refine culturally tailored intervention content for Hispanic adult subgroups.

**Conclusions:**

This formative pilot study will inform the cultural adaptation and refinement of an mHealth app, VITAL, which will be tested in a subsequent randomized controlled trial to improve health disparities and assist Hispanic people with HIV in quitting smoking.

## Introduction

People with HIV/AIDS are at increased risk for smoking, with smoking rates more than double those of the general population (33.6% [1] vs 12.5% [2]). People with HIV are less likely to quit smoking [1,3], likely due to the complex interplay among co-occurring behavioral risk factors and limited resources [4,5]. As a result, people with HIV who smoke experience more tobacco-related disease and illness compared with adults without HIV who smoke [6]. In fact, people with HIV now lose more life-years to tobacco use than to HIV [7], and almost one-quarter of all deaths among people with HIV on antiretroviral therapy (ART) are attributed to smoking [8]. Ethnic minority status can play a role in observed health inequities through social, economic, environmental, and structural disparities at the intrapersonal, interpersonal, institutional, and systemic level [9]. For example, Hispanic people represent 18% of the population but accounted for 32% of new HIV diagnoses in 2022 [10]. Relative to White individuals with HIV, fewer Hispanic adults with HIV receive HIV care (79% vs 74%), report ART adherence in the past month (67% vs 57%), and reach viral suppression (71% vs 65%) [11]. Regarding smoking, although prevalence rates are lower among Hispanic persons relative to the national smoking rate (9.8% vs 13.7%) [12], and Hispanic people who smoke are more likely to be intermittent smokers and smoke fewer cigarettes per day compared with non-Hispanic White individuals [13], smoking-related disease and illness is the leading preventable cause of morbidity and mortality among the Hispanic community in the United States [14]. From an intersectional stigma and discrimination perspective [15] and socioecological models of social determinants of health [16], Hispanic adults living with HIV who smoke may be at elevated risk for worse HIV disease management and smoking outcomes and may ultimately experience increased health disparities [5].

Hispanic adults with HIV experience greater stressors [17], including stigma and discrimination, that result from the intersection of ethnic minority status [18] and HIV status [15]. These factors affect both mental and physical health functioning [19,20] and contribute to maladaptive coping strategies, including smoking [19]. Emerging evidence suggests it may be beneficial to target core mechanisms implicated in stress responses and smoking behavior as an integrated method to support smoking cessation. A prominent construct to consider within this area of research is anxiety sensitivity (AS) [21]. AS reflects the fear of symptoms that accompany anxiety [22]; it has demonstrated racial and ethnic invariance [23]. Among Hispanic adults in general, AS serves as a mechanism between perceived racial discrimination and depression, social anxiety, anxious arousal, and mood and anxiety disorders [24]. Moreover, AS has been associated with worse smoking cessation outcomes (ie, cigarette dependence, perceived barriers for quitting, and quit problems) among this group [25]. Additionally, people with HIV who smoke often experience elevated AS and have higher average levels [26,27] than the general population [28]. Given these relations and evidence that HIV-related stress and mood management are leading barriers to smoking cessation among this group [29], AS may serve as a potential mechanism to target in smoking cessation and HIV care management among Hispanic people with HIV who smoke.

AS can be effectively targeted through psychoeducation, cognitive restructuring, and interoceptive exposure exercises [30]. Among people with HIV, improvements in AS following treatment are related to decreased anxiety and depressive symptoms, as well as overall better quality of life [31-33]. Such improvements may support better HIV-specific outcomes, including ART adherence [34] and treatment engagement [35]. Among people with HIV who smoke, integrated in-person AS reduction and smoking cessation programs developed by our team have resulted in improved smoking cessation outcomes [36]. AS can be successfully addressed using in-person and digitally delivered methods [30]. These findings are clinically important, as a digitally delivered AS reduction program may be more appealing to Hispanic people with HIV who smoke, given that it would be easily accessible, remove logistical barriers to treatment, be tailored to their unique needs, eliminate considerable stigma associated with accessing treatment, and be adaptable to their language preference [37]. Importantly, technology-based (eg, mobile health [mHealth]) HIV treatment adherence interventions have been shown to improve ART adherence, viral load suppression, and engagement in care among people with HIV [38]. Additionally, initial evidence supports mobile phone–based smoking cessation interventions (largely text message–focused) for the general population and among people with HIV [39].

Against this background, members of the current research team have developed and tested a culturally tailored mHealth intervention for smoking cessation, AS reduction, and HIV care improvement among Black people with HIV who smoke [40]. The program (Mobile Anxiety Sensitivity Program for People With HIV; MASP+) was developed using an iterative, community-informed design process that built on prior MASP (Mobile Anxiety Sensitivity Program) development and integrated mHealth intervention work [41,42]. The intervention is grounded in the AS framework, which conceptualizes fear of internal sensations as a transdiagnostic mechanism contributing to smoking maintenance and HIV-related health behavior challenges [43,44]. Intervention components draw on evidence demonstrating that AS reduction strategies, including psychoeducation and interoceptive exposure, can improve smoking-related outcomes [45-47]. Development of MASP+ involved systematic cultural adaptation of existing MASP content guided by socioecological and minority stress frameworks, which emphasize the role of structural, interpersonal, and stigma-related factors in shaping smoking and HIV outcomes [16,48]. Adaptation followed culturally responsive intervention design principles, including modification of language, imagery, and examples to reflect the lived experiences of Black adults with HIV, integration of content addressing discrimination and structural inequities, and incorporation of stakeholder feedback from subject matter experts and a Community Research Advisory Board (CRAB) [49-51].

Together, this prior work establishes MASP+ as a theoretically grounded, culturally tailored mHealth intervention framework targeting smoking, AS, and HIV care [41,42]. Disparities and cultural adaptation frameworks indicate that such interventions require population-specific refinement to reflect differences in sociocultural context, language, and minority stress processes that influence smoking and HIV outcomes, particularly among groups that experience health disparities [16,49,50]. Hispanic adults with HIV who smoke experience distinct structural, linguistic, and sociocultural influences that are not explicitly addressed in MASP+ and experience significant smoking-related and HIV-related health disparities. Accordingly, the proposed work will refine, adapt, and culturally tailor existing AS reduction and smoking cessation intervention materials for Hispanic adults to enhance cultural relevance, address population-specific mechanisms contributing to disparities, and improve accessibility through linguistic adaptation, thereby reducing disparities in smoking cessation and HIV care engagement. The objectives of this study are to (1) culturally adapt and translate intervention content for Mexican/Mexican American, Central American, and South American adults with HIV who smoke and (2) evaluate and refine intervention content based on end-user feedback. The goal of this process is to develop linguistically and culturally specific versions of intervention content for these subgroups to inform the future development of VITAL, an mHealth app. Specific details of the study objectives are as follows:

1. To adapt and culturally tailor intervention materials in English and Spanish for Mexican/Mexican American, Central American, and South American adults with HIV who smoke cigarettes based on information from current literature, study team expertise, and feedback from the CRAB at the University of Houston.
2. Enroll 80 participants who identify as Mexican/Mexican American, Central American, or South American; currently smoke; and report living with HIV to review and provide feedback on intervention content. Each group will have its own version of the intervention content.

## Methods

### Objective 1

Members of the current research team previously developed an mHealth intervention for AS reduction, smoking cessation, and HIV care engagement and management for Black adults with HIV who smoke (MASP+) [40]. The current team used these materials and culturally adapted them for Mexican/Mexican American, Central American, and South American adults with HIV who smoke to inform the future development of a similar mHealth app, VITAL. Specifically, MASP+ treatment video scripts were reviewed and adapted based on shared cultural considerations particular to the target populations. Additional materials were created as needed based on published literature relevant to the specific population’s stressors, such as deportation concerns or a strong emphasis on family connections. Adapted content from MASP+ content included information related to standard smoking cessation treatment, psychoeducation on AS, information on HIV care (including methods to improve symptom management, medication adherence, and treatment engagement), the role of racism, discrimination, and stigma in health care, interoceptive exposure exercises, and social support. In addition, new content was developed specifically for the priority population, focused on health disparities related to smoking and HIV among Hispanic adults. The integration of modified and new content ensured discussion of the intersection of these topics as they related specifically to Hispanic adults with HIV who smoke. Further, all content was updated to reflect linguistically appropriate and culturally representative examples; aspects of the adaptation process contributed to the development of 3 versions of linguistically unique materials, each tested on a specific subgroup. Content was made available in both Spanish and English.

We followed the 4-phase process of Barrera and González Castro heuristic framework for the adaptation process, which includes (1) information gathering, (2) preliminary adaptation design, (3) preliminary adaptation tests, and (4) adaptation refinement [52]. The minority stress model [53] (as related to ethnic minority status and HIV status) and the sociocultural model for addiction among Hispanic adults [54] also guided material modification and development. Following adaptation and development of new materials by the research team, the CRAB at the University of Houston reviewed the materials and provided written feedback, which was discussed and implemented by the research team.

Intervention content (ie, scripts) had been drafted using a cultural considerations document developed by the current research team that listed cultural considerations based on extensive literature [54-56]. The cultural considerations document integrated research on Hispanic culture and behavioral determinants from multiple disciplines, including public health and psychology, as well as review and input from community practitioners treating Hispanic people with HIV who smoke. After the literature search was complete, previously developed scripts for African American individuals who smoke and are living with HIV were edited and adapted for the Hispanic subgroups using the cultural considerations document. The cultural considerations document addressed personalized concerns and stressors that may impact Mexican/Mexican American, Central American, and South American individuals’ smoking cessation outcomes and HIV care management.

The scripts (20 total) were then reviewed by the research team and presented to the CRAB for further feedback. Finalized versions of the English intervention content (ie, scripts) were then translated into Spanish by a certified professional translation service. To ensure the accuracy of translations, a robust methodology was employed to ensure linguistic equivalency, including back-translations by the research team. Further, the Spanish scripts were subsequently reviewed for regional linguistic variations (eg, “popote” [used in Mexico] and “pitillo” [common in South America] as different terms for “straw”) by a study team member who self-identified with the relevant Hispanic subgroup for each corresponding video. Following the back-translation, scripts were checked against the English versions to ensure the same content was conveyed.

Once all scripts were finalized, the research team used Murf.AI [57] to create audio files of the scripts. Voices selected to read the scripts were a male and a female adult who identified as Hispanic. Murf.AI–generated audio files (both English and Spanish versions) were then reviewed by the research team to check for accuracy, word pronunciation, and speaking tempo. Spanish audios were reviewed by a member of the study team who identified as part of the Hispanic subgroup of the audio. Final edits were then applied to the audio files based on the research team’s feedback. The final audio files were integrated into the created videos.

Videos were created using the animated video creation platform Vyond [58]. Consistent with previous work [40], once a video was drafted, it was reviewed by an additional member of the research team for feedback before being sent to the coinvestigators for review. Video feedback focused on the appropriateness of the animations, cultural appropriateness, timing with Murf.AI audio files, and whether the overall creation reflected Mexican/Mexican American, Central American, and South American groups. Updated videos were then presented to the CRAB for feedback. Written feedback from CRAB members was distributed to the research team and appropriately implemented.

To ensure that participants would view the same content regardless of language preference, Spanish videos were created from copies of the finalized English videos for each subgroup. The tailored Spanish audio files for each subgroup were then uploaded into Vyond, and members of the research team adjusted the timing of the animations to match the Spanish audio. The translation feature in Vyond was used to translate all English words in the videos into Spanish. Members of the research team who were fluent in Spanish checked the accuracy of the translations. Spanish videos were then reviewed by an additional member of the research team before being reviewed by a Hispanic expert for feedback and finalization. A total of 40 videos (20 English and 20 Spanish) were created for each subgroup.

Additional study materials (measures, consent form, Health Insurance Portability and Accountability Act authorization confirmation texts and emails, and standard operating procedure scripts) followed a similar translation process as the intervention content (ie, scripts). Study materials, including measures without a Spanish version, were translated into Spanish by a professional organization. Following the translation, all materials were then back-translated and checked for accuracy by a member of the research team.

### Objective 2

#### Participant Eligibility

The study eligibility criteria included (1) being at least 18 years of age; (2) reporting daily smoking or smoking at least 10 cigarettes per week; (3) providing a current picture of their cigarette package for remote verification of smoking status [41]; (4) self-identifying as Hispanic (Mexican/Mexican American, Central American, or South American); (5) living with HIV (confirmed via electronic medical chart review or self-report); (6) elevated AS, as indicated by a score of 5 or higher on the Short Scale Anxiety Sensitivity Index (SSASI), which is consistent with prior research among people with HIV [59]; and (7) being motivated to quit smoking (ie, rating their motivation to quit smoking at least a 5 out of 10). Exclusion criteria included (1) an inability to provide informed, voluntary, written consent to participate and (2) not agreeing to be audio recorded.

#### Procedures

Mexican/Mexican American, Central American, and South American participants were recruited from a large, academically affiliated safety-net clinic for people with HIV via flyers placed throughout clinics and referrals from clinicians. Recruitment flyers were also posted on online community pages, around the Houston area (eg, bus stops, community centers), and on various social media platforms (eg, Facebook, Craigslist, Reddit, Twitter). Participants were also recruited from BuildClinical, a research advertising tool that assists with participant recruitment by deploying advertisements across web platforms to target and engage specific populations through its patient advertising network. Interested individuals completed an online screener through Qualtrics, an online survey administration system, which could be accessed using a QR code or a short URL on the study flyer. The online prescreener was used to assess initial eligibility. To ensure data validity, all responses were checked after the prescreener survey was completed. Validation of participant responses included checking for a valid phone number, geolocation within the United States, duplicate IP addresses, and prior ineligible submissions.

**Figure 1.**
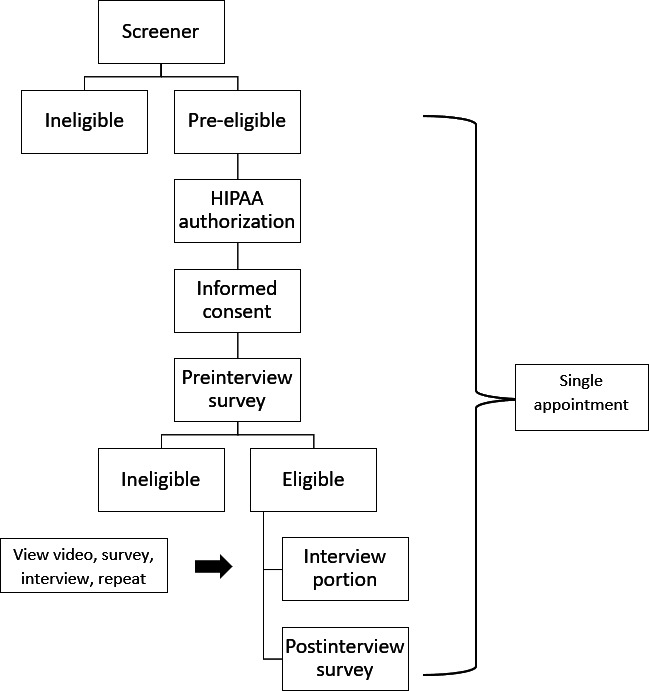
Participant flowchart. HIPAA: Health Insurance Portability and Accountability Act.

Participants eligible at the prescreener were prompted to select a date and time to complete a 2-hour remote interview appointment conducted via Zoom. In-clinic assisted enrollment and interview procedures were also available at the Thomas Street Clinic at Quentin Mease Health Center in Houston, TX. At the interview appointment, trained research staff first collected informed consent to confirm eligibility. The consent process included informed consent, a Health Insurance Portability and Accountability Act consent form, HIV status verification (via showing the verification to the camera for the study team member to verify or uploading of ART, medical report/record of HIV diagnosis, or HIV viral load assessment), and smoking status verification (providing a current picture of their cigarette package). During the consent process, participants’ geolocation was checked to confirm that they were in the United States during the appointment as an additional validation measure. Participants eligible for the study then completed a self-report preinterview assessment, an interview to provide feedback on the intervention materials, and a self-report postinterview assessment (Figure 1). Study appointments were conducted in Spanish or English based on participant preference. Following completion of the postinterview survey, participants received a US $100 electronic gift card for participation.

During the interview, participants reviewed 4 (out of 20) culturally tailored videos (3‐5 min each) in the language of their preference (Spanish or English) that were linguistically specific to their subgroup. The 20 videos for each language and subgroup were divided into 5 groups of 4 videos based on the overall topic of the video (Stress, Nicotine Replacement Therapy, Flexible Thinking, Mindfulness, and Managing Uncomfortable Sensations). Qualtrics randomly assigned which group of videos each participant viewed during the interview. A quota system was used so that each group of 4 videos was removed from the rotation after being viewed 7 times. Although some videos may reference content in other videos, the materials were appropriate to be reviewed on their own. Following each video, participants completed a brief survey on content relevance, appropriateness, ease of understanding and clarity, usefulness, and helpfulness [60]. After completing the survey, research staff followed a semistructured interview guide to assess video impressions, identify opportunities to improve content tailoring, gather suggestions for additional topics to include, and assess the relevance of the content. Semistructured interview guides also explored how sociocultural factors may be appropriately integrated into the content (eg, potential interpersonal, stigma, social, family, and religious considerations). The interview portion for all participants was recorded, and after the interview was completed, the audio file was transcribed and translated using a professional transcription and translation service. Following transcriptions, each video recording was deleted.

Study team members attended multiple training sessions on qualitative interviewing and received ongoing supervision. During the training, research staff reviewed how to conduct a semistructured interview and how to effectively probe participants to respond to questions without guiding them toward specific answers in the materials. After the training sessions, members of the team were instructed to practice with each other and prepare questions and personal feedback to discuss during meetings. Additional quality assurance and fidelity measures included a qualitative researcher’s review of initial interviews, a midpoint discussion to review selected interviews, and, as needed, discussion of challenging interviews with individual interviewers.

#### Measures

Table 1 summarizes the measures used for Objective 2, including their corresponding survey time points across the study. Measures span demographics, mental health, smoking, HIV-related variables, and intervention acceptability, with administration occurring from prescreener through postinterview assessments.

**Table 1.** Objective 2 measures.

	Items	Prescreener survey	Preinterview survey	Interview survey	Postinterview survey
Demographics and background					
Demographic questionnaire^a^	24	✓	✓		
Mental health variables					
Short Scale Anxiety Sensitivity Index^a^ [59]	5	✓			
Smoking variables					
Smoking History Questionnaire^b^ [61]	18		✓		
Motivation to quit^a^	1	✓			
Heaviness of Smoking Index^b^	2	✓			
HIV-related variables					
HIV status and history^b^ (self-report or chart review)	1	✓			
AIDS Clinical Trials Group adherence questionnaire^b^ [62]	10		✓		
Index of engagement in HIV care^b^ [63]	10		✓		
Intervention acceptability variables					
Perception of intervention content^c^ [60]	6			✓	
Qualitative interview	24			✓	
Credibility/expectancy questionnaire^d^ [64]	6				✓
Advertisement attitudes measure^d^ [65]	5				✓
Iowa Cultural Understanding Assessment-Client Form^d^ [66]	15				✓

a Eligibility or screening.

b Preinterview descriptors.

c Per-video ratings.

d Postinterview global measures.

##### Demographics and Screener Questions

Participants were asked to provide standard demographic information (ie, name, contact information, age, sex, race, ethnicity, brief smoking history, etc). Participants also provided their language preference. Select items were used as eligibility criteria. Name and contact information were used for scheduling and interview purposes and will not be part of the dataset.

##### Anxiety Sensitivity

AS was assessed using the SSASI [59]. The SSASI is a 5-item measure that was used to provide a descriptive assessment of AS at prescreener by using a 5-point Likert scale (0=very little to 4=very much). The SSASI was used as the AS eligibility criteria at prescreener.

##### Smoking Behavior

Smoking behavior was collected during the preinterview. The Smoking History Questionnaire [61] is an 18-item self-report questionnaire used to assess smoking history, pattern, and use of evidence-based pharmacological and nonpharmacological cessation treatment (“Since you started regular smoking, what is the average number of days per week you smoke?”). This measure was used to evaluate an individual’s current smoking habits.

##### Motivation to Quit

Motivation to quit smoking, an eligibility criterion, was assessed at the prescreener using the following question: “On a scale from 0 to 10 with 0 being ‘not at all motivated’ and 10 being ‘extremely motivated’, how motivated are you to quit smoking in the next month?”

##### Nicotine Dependence

The Heaviness of Smoking Index [67] measures nicotine dependence and the intensity of nicotine addiction. Participants answered 2 questions based on their smoking habits (“On days that you smoke, how soon after you wake up do you smoke your first cigarette?” and “How many cigarettes do you typically smoke per day?”). This measure was collected at preinterview for descriptive purposes.

##### HIV Status and History

Participants answered whether they had ever been diagnosed with HIV and when they were first diagnosed with HIV at the prescreener to determine eligibility. HIV status was reported via chart review if the participant was a clinic patient or via self-report. If self-reporting, participants presented their ART medication with their name and the medication name visible, documentation of their diagnosis, or their most recent viral load assessment.

##### HIV Treatment–Related Variables

The AIDS Clinical Trials Group Adherence Questionnaire [62] assesses ART adherence and reasons for missed medication and nonadherence across 20 items (“How sure are you that your HIV management medication will have a positive effect on your health”). HIV treatment engagement was assessed using the Index of Engagement in HIV Care [63], a 10-item scale that asked participants about their experience of HIV care (“How much do you trust your HIV care provider?”). HIV treatment–related measures were collected at preinterview and will be used for descriptive purposes.

##### Qualitative Interview

The semistructured qualitative interview assessed ways to improve the animated videos and scripted content (Multimedia Appendix 1). The question path was developed based on previous formative interview question paths [40], then modified to assess video content for cultural relevance, ease of understanding, and to solicit input for additional content to increase clarity and acceptability. The question path was tested prior to implementation. Participants first completed a set of quantitative questions from the Perception of Intervention measure, which was adapted from prior work [60]. The Perception of Intervention Content measure was administered immediately after each video and prior to the video-specific qualitative interview questions. The Perception of Intervention Content measure assessed quantitative ratings of personal relevance, clarity of understanding, perceived helpfulness for understanding smoking-stress and smoking-HIV links, and motivation to improve HIV care and reduce smoking. These immediate postvideo ratings capture participants‘ initial reactions and will be used to identify video segments that may require targeted refinement. Qualitative interview questions (e.g., “What could we change about this video to make it more interesting or more motivating for you?”) were asked following the Perception of Intervention Content measure, and statements to which participants responded “Strongly Disagree,” “Neither Agree nor Disagree,” or “Disagree” were probed during the qualitative interview. For example, “I see that you responded neutral or disagreed with the statement ‘(repeat the statement they disagreed with)’. Tell me about your ‘neutral‘ or ‘disagree’ response here. I’d like to understand that.” Finally, participants were asked a set of qualitative questions to gather their impressions of all 4 videos as a group (eg, “Can you think of an example where one of the videos talked about smoking or HIV in a way that fit with [Mexican/Mexican American, Central American, South American] culture?”).

##### Intervention Acceptability Variables

The interview and postinterview survey were used to assess intervention acceptability. The Credibility/Expectancy Questionnaire [64] is a 6-item measure regarding perceptions of treatment credibility and expectancies about treatment, along with acceptability and satisfaction (“At this point, how successful do you think the app content would be in helping you quit smoking?”). Moreover, the Advertisement Attitudes Measure [65] is a 5-item subscale that assessed intervention relevance (“The videos were meaningful.”). Finally, the Iowa Cultural Understanding Assessment-Client Form [66], a 15-item measure evaluating the intervention’s cultural competence, was provided at postinterview.

### Data Analysis

#### Quantitative Data

Quantitative analyses will focus on descriptive statistics (means, SDs, and response distributions) for intervention acceptability measures collected at 2 levels. Video-specific acceptability will be assessed using the Perception of Intervention Content measure, administered immediately after each video and prior to the video-specific qualitative interview questions. The Perception of Intervention Content measure ratings will be summarized at the item and video levels to identify patterns in perceived relevance, comprehension, and motivational impact across intervention components and to identify specific video segments that may require refinement. Global postinterview acceptability will be assessed using the Credibility/Expectancy Questionnaire, Advertisement Attitudes Measure, and Iowa Cultural Understanding Assessment-Client Form. These measures capture overall perceptions of intervention credibility, satisfaction, perceived effectiveness, relevance, and cultural competence across the intervention as a whole. Quantitative acceptability findings will distinguish between video-specific refinement needs (informed primarily by the Perception of Intervention Content measure ratings) and broader intervention-level acceptability considerations (informed by global postinterview measures). Quantitative acceptability ratings will be used to identify specific and overall intervention components demonstrating lower perceived clarity, relevance, usefulness, credibility, or cultural appropriateness that may warrant refinement.

#### Qualitative Data

After completion of the semistructured interviews, transcriptions were reviewed by the research team and assessed for saturation of concepts. Thematic analysis will be conducted following Braun and Clarke [68]. Qualitative data captured participant perspectives on cultural relevance, clarity, engagement, linguistic appropriateness, perceived usefulness, and suggested modifications for each video and for the intervention overall. Qualitative interview questions followed completion of the Perception of Intervention Content measure for each video, allowing participants’ narratives to be interpreted in relation to their quantitative ratings. Attention will be given to qualitative explanations corresponding to the Perception of Intervention Content measure items with lower ratings in analyses, as these provide direct guidance for targeted content modification. The multidisciplinary qualitative team will develop the codebook and then code the transcribed interviews in 2 coding passes: first by interview question and then for higher-level concepts and theoretical constructs identified in the first coding pass. The 2 interview coders will code 2 interviews independently and then review them with the qualitative researcher (MKC). Any discrepancies will be discussed and revised as needed. If there is good correspondence, the remaining interviews will be divided between the 2 coders, coded independently, and then reviewed by the other coder. Finally, the qualitative researcher will review the coding, and the team will meet to discuss and reach consensus on any areas of disagreement. Coded transcripts will then be analyzed for themes and subthemes. Next, the thematic analysis process will be reviewed by another member of the team to assess the trustworthiness of the data analysis process and review the coded transcripts for confirming and disconfirming evidence of themes. The themes and subthemes will be triangulated with quantitative data and then analyzed by geographic subgroup, as applicable.

#### Mixed Methods Integration (Triangulation)

**Figure 2.**
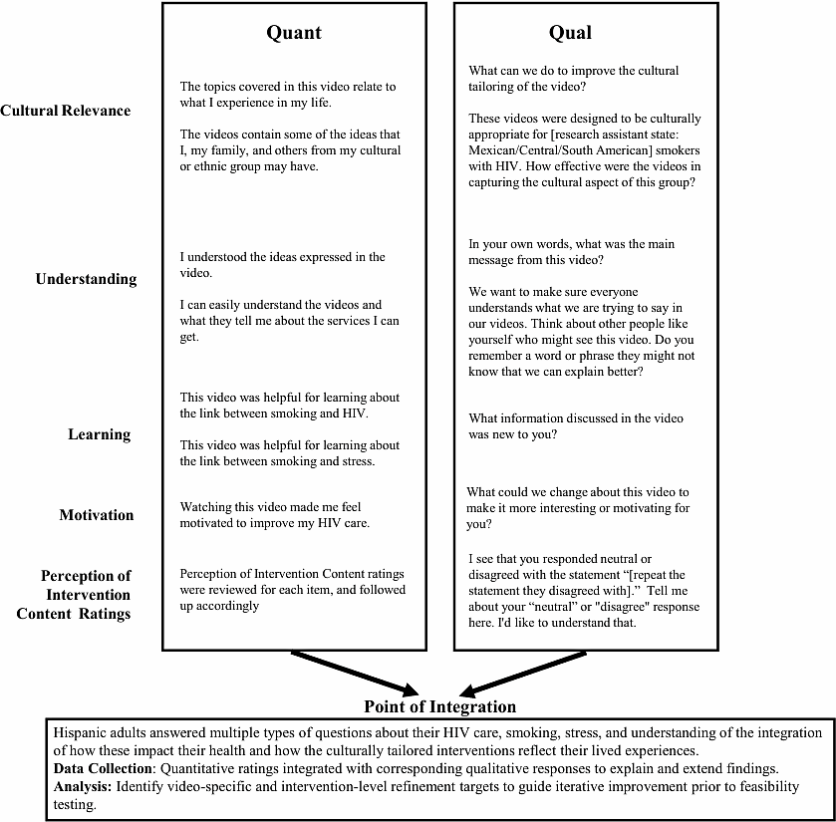
Mixed methods integration framework.

A triangulation mixed methods data analysis approach [69,70] will be used to integrate quantitative and qualitative findings to guide intervention refinement (see Figure 2). Points of integration will be identified through alignment of corresponding qualitative interview questions and quantitative measures, followed by identification of additional conceptual integration points. Integration will occur at the level of video content and thematic domains and will examine qualitative themes alongside quantitative ratings to identify convergence, complementarity, or divergence across data sources.

Lower Perception of Intervention Content ratings will be examined alongside participants’ explanations to identify specific content elements contributing to reduced clarity, relevance, or motivational impact. Global acceptability measures will contextualize overall perceptions of intervention credibility, usefulness, and cultural appropriateness across intervention materials. For example, videos receiving lower quantitative ratings (eg, clarity or cultural relevance) will be reviewed alongside corresponding qualitative feedback to determine specific revision targets.

Integrated findings will be synthesized into actionable revision targets, with priority given to content areas showing convergence between lower quantitative ratings and recurrent qualitative suggestions. Content demonstrating consistently favorable ratings and minimal suggested changes will be retained. Integrated results will directly inform iterative refinement of intervention materials and determination of readiness for subsequent feasibility and implementation testing.

### Sample Size Determination

A total of 80 participants were enrolled to ensure a sufficient sample for identifying patterns across diverse Hispanic subgroups and to reach thematic saturation in qualitative analysis [71]. Recruitment across Mexican/Mexican American, Central American, and South American participants was intended to be approximate and guided by local clinic demographics and recruitment feasibility rather than fixed subgroup targets (a maximum of 35 and a minimum of 10 per subgroup). Recruitment was monitored throughout the study to support representation across subgroups, and outreach could be prioritized for underrepresented groups as needed to obtain sufficient feedback to inform linguistic tailoring. This distribution reflected the relative prevalence of these subgroups in the study’s recruitment areas and enabled meaningful, culturally specific feedback while maintaining feasibility for a pilot study. Representation across subgroups strengthened the cultural validity of the intervention materials and supported tailored refinement decisions based on thematic saturation and relevance of feedback rather than equal subgroup sample sizes.

### Ethical Considerations

This study was funded in 2023 by the Research Centers in Minority Institutions program of the National Institute on Minority Health and Health Disparities (3U54MD015946-04S3). The University of Houston Institutional Review Board (STUDY00004409) reviewed the study, provided ethical approval, and confirmed that all study procedures complied with applicable federal regulations. Checklist 1 presents the Standards for Reporting Qualitative Research (SRQR) checklist, which was used to guide the current reporting and ensure comprehensive and transparent presentation of methods.

Individuals were required to provide informed consent prior to participation. All procedures were conducted electronically and included comprehension of the study’s purpose, procedures, potential risks, and benefits.

Consent materials and survey data are stored within the Qualtrics platform, a secure platform designed to safeguard participant information. Only the research personnel who are part of the study team can access study data using password-protected devices with 2-factor authentication. Participant identifiable information (eg, names and email addresses) will not be included in any reports or publications, as participants are identified in the dataset using only unique study identification numbers.

Participants were required to upload documentation confirming HIV status (eg, ART, medical records of diagnosis, or HIV viral load results) and cigarette use verification (eg, used or unused cigarettes or a pack of cigarettes) for study eligibility. Upon study completion, participants received a total of US $100 in electronic gift cards for completing the preinterview survey, the interview, and the postinterview survey. The compensation amount was determined based on the time and effort it took for participants to complete all study components.

## Results

### Timeline

Objective 1 was completed in August 2024. Materials were linguistically tailored to each geographic subgroup, but the content was the same. Data collection for Objective 2 began on August 19, 2024, and was completed on June 26, 2025. Data cleaning, mixed methods data analysis, and content refinement are anticipated to be completed by September 2026. Quantitative ratings of acceptability and qualitative interview feedback will be integrated using a triangulation approach to identify priority adaptation targets, including areas of lower relevance, clarity, cultural fit, and perceived usefulness.

Integrated findings will guide systematic, subgroup-specific refinement of intervention content for Mexican/Mexican American, Central American, and South American adults with HIV who smoke. This process will result in finalized English and Spanish culturally and linguistically tailored video libraries for each subgroup, along with documentation of adaptation decisions and identified refinement targets.

Refined materials will be prepared for integration into the VITAL mHealth app to support subsequent evaluation of feasibility, acceptability, and potential efficacy for smoking cessation and HIV care engagement among Hispanic adults with HIV who smoke.

### Study Status

Objective 1, which included the development of culturally tailored treatment interventions, study document translations, and CRAB review, and part of Objective 2 (recruitment and data collection) were completed before manuscript submission. This project was funded in May 2023. Data cleaning, analysis, and content refinement are currently underway. This protocol is not prospectively registered, as this study was designed as a pilot formative study. See Textbox 1 for the study workflow diagram.

Textbox 1. Study workflow diagram. The asterisk (*) indicates activities that have been completed.

**Phase 1: Preliminary cultural adaptation**

1. Information gathering and content foundation*

- Mobile Anxiety Sensitivity Program (MASP+) scripts were used as an evidence-based foundation for adaptation.
- Literature review and team expertise → cultural considerations document.
- Identification of subgroup-specific sociocultural factors (Mexican/Mexican American, Central American, South American).

2. Preliminary adaptation design*

- Theory-informed cultural and linguistic adaptation of MASP+ scripts.
- Research team script review and refinement.
- Script review by Community Research Advisory Board (CRAB) for further feedback.
- Professional translation → Spanish scripts.
- Linguistically tailored review for regional variation.
- English and Spanish script review for content accuracy.
- Development of animations in both Spanish and English.
- Video reviews by the research team for content accuracy.
- Video review by CRAB for further feedback.

**Phase 2: Protocol for cultural adaptation testing**

1. Preliminary adaptation testing*

- Finalized video sets were prepared for formative evaluation.
- Development of interview guide and acceptability measures.
- Research staff completed training and supervision in qualitative interviewing.
- Participants completed a self-report preinterview.
- Participants completed a qualitative interview to provide feedback on intervention materials.
- Participants completed a postinterview survey.

2. Adaptation refinement

- Mixed methods feedback synthesis (qualitative and quantitative).
- Codebook development → 2-pass thematic coding.
- Identification of priority adaptation targets.
- Integration of quantitative and qualitative findings to guide revisions.
- Subgroup-specific refinement of intervention content (as appropriate).
- Final culturally and linguistically tailored materials for VITAL integration.
- Findings inform subsequent mHealth application development (VITAL).

## Discussion

### Implications and Next Steps

Hispanic adults with HIV who smoke cigarettes in the United States represent a group facing health disparities, leading to worse smoking and HIV outcomes [5], posing a major public health concern. This study is the first to develop culturally adapted content that seeks to improve smoking cessation and HIV outcomes among unique subgroups of the Hispanic community (Mexican/Mexican American, Central American, South American). Currently, there are no integrated interventions that seek to simultaneously improve smoking and HIV outcomes in a culturally sensitive way for Hispanic adults. By integrating culturally relevant content to target a known risk factor for worse smoking cessation and HIV outcomes (ie, AS), this study has the potential to develop appropriate content that can be easily integrated into an accessible, effective, potentially low-cost smartphone-based intervention. This intervention could improve smoking cessation and HIV care engagement, HIV-specific quality of life, and medication adherence among Hispanic adults with HIV who smoke. Further, through the process of piloting content, we will develop complementary materials tailored to the unique cultural backgrounds of Hispanic participants. The content of the app will address known barriers to HIV care engagement, ART adherence, and utilization of smoking cessation services among Hispanic adults, including access, affordability, cultural appropriateness, real-time support, and stigma, with the goal of improving HIV-related health outcomes and reducing health disparities among the Hispanic community.

Clinically, integrating AS reduction skills and cultural tailoring into the intervention material represents a groundbreaking approach. Experiences of AS and related mental health factors (eg, anxiety, depression, and drug withdrawal or craving) can vary over time and among individuals [72]. AS reduction improves anxiety, depression, and enhances quality of life among people with HIV [31-33], which can improve HIV-specific outcomes, including ART adherence [34] and treatment engagement [35]. Smoking cessation treatment that specifically targets AS has led to better smoking outcomes [73]. However, no work has leveraged the potential of targeting AS reduction to improve smoking cessation and HIV care outcomes in the context of an accessible, tailored intervention for Hispanic adults living with HIV who also smoke. This innovative approach may serve as a potential solution to address the documented health disparities experienced by Hispanic adults with HIV who smoke. Additionally, the tailored intervention materials, available in both Spanish and English, address factors that impede successful behavioral change in a culturally appropriate, person-centered manner. This could have strong implications for mitigating health disparities in Hispanic adults with HIV who smoke.

### Limitations

This study has several limitations. The focus on only 3 subgroups of the larger Hispanic population (Mexican/Mexican American, Central American, South American) does not allow for specialized culturally tailored treatment for the broader heterogeneous Hispanic population. Although the 3 subgroups represent Hispanic adults from similar regions, individuals within each region may have unique cultural experiences that may not be fully represented in the intervention materials. Additionally, despite in-clinic assisted enrollment efforts, some degree of selection bias toward more resourced participants may persist. Finally, the study is not a randomized controlled trial (RCT). Due to the novelty of the tailored intervention content, an RCT would be premature before receiving feedback on the cultural appropriateness and relevance of the materials.

### Conclusion

The goal of this study is to develop culturally tailored content for smoking cessation, HIV care, and AS reduction, specifically developed for the cultural backgrounds of 3 subgroups in Hispanic communities (Mexican/Mexican American, Central American, South American). The updated, culturally tailored intervention materials have the potential to address the mental and physical barriers to smoking cessation and treatment engagement that are unique to Hispanic adults with HIV who smoke. Future work will focus on implementing the culturally tailored intervention materials into the VITAL app for delivery via an mHealth intervention, which will then be tested in a larger RCT, with the goal of potential dissemination of intervention materials. This strategic approach is consistent with the need to address HIV-related disparities among Hispanic adults, which outlines the need for improving treatment accessibility, developing tailored behavioral interventions, and recognizing community diversity [74]. By also addressing smoking, a risk factor for worse HIV outcomes, this study aims to reduce health disparities within the Hispanic community and advance accessibility to care.

## Supplementary material

10.2196/81192 Multimedia Appendix 1 Semistructured interview guide.

10.2196/81192 Checklist 1 SRQR (Standards for Reporting Qualitative Research) checklist [75].

## Abbreviations

ART: antiretroviral therapy
AS: anxiety sensitivity
CRAB: Community Research Advisory Board
MASP: Mobile Anxiety Sensitivity Program
MASP+: Mobile Anxiety Sensitivity Program for People With HIV
mHealth: mobile health
RCT: randomized controlled trial
SSASI: Short Scale Anxiety Sensitivity Index
