# Supplementary material for: Culturally Tailored Materials for Smoking Cessation and HIV Care Adherence Among Hispanic Adults: Protocol for Content Development and Formative Evaluation
Source: JMIR Res Protoc. 2026 Apr 29;15:e81192. doi: 10.2196/81192 (PMC13128065; doi:10.2196/81192)
Supplement: Multimedia Appendix 1 [file resprot-v15-e81192-s001.docx]

**Multimedia Appendix 1: Semi-Structured Interview Guide**

***Content Improvement Interview***

The Content Improvement Interview is a qualitative assessment that was used to assess participant experiences with application content. This interview was conducted with each participant and was recorded for qualitative data analysis. This interview was modified from a survey created by Casey and colleagues *(Casey et al., 2014).*

***Note to Interviewer on Interview Structure***

Videos were presented to participants one at a time. After each video, participants answered a series of quantitative questions on their own via Qualtrics. Once they were done, the interviewer asked additional open-ended questions. The videos covered similar topics, so after the 4 videos were played, participants answered some additional questions about the group of videos as a whole. After watching the final video, we asked participants some additional questions to gauge their overall impressions. This included a final activity in which a screenshot representing each video watched was displayed.

***Introduction***

The current interview for VITAL is with participant number [*state their PT identification number*]. Today is [*state today’s date including the year*]. The interview is being conducted by [*Your first and last name*].

Thank you for agreeing to take part in this discussion today. My name is [*your name*] and I work with the University of Houston. I am going to show you several different short videos and would like to get your impressions of each of the videos. There are no right or wrong answers to my questions, I am just interested in getting your impressions and learning how the videos can be improved so people will be more likely to use them and they are easier to understand. I was not directly involved with the creation of these materials. My role is simply to obtain your honest feedback.

We record these discussions so we can fully focus on the experiences you had watching the videos. After the call is complete, the interview will be typed up and the audio file will be deleted. This discussion is confidential and no names will be used in our report. Do you have any questions about this? Do you consent to having this conversation recorded?

Are you ready to begin?

To get us started, I will play a video then ask you about your overall impressions

[*RA plays the first video selected for the participant to watch*]

***Video X Quantitative Questions***

| Statement | Strongly Disagree | Disagree | Neither Agree nor Disagree | Agree | Strongly Agree |
| --- | --- | --- | --- | --- | --- |
| 1. The topics covered in this video relate to what I experience in my life. | — | — | — | — | — |
| 2. I understood the ideas expressed in the video. | — | — | — | — | — |
| 3. This video was helpful for learning about the link between smoking and stress. | — | — | — | — | — |
| 4. This video was helpful for learning about the link between smoking and HIV. |  |  |  |  |  |
| 5. Watching his video made me feel motivated to improve my HIV care. | — | — | — | — | — |
| 6. Watching his video made me feel motivated to cut down or stop smoking. | — | — | — | — | — |

***Video X Interview Questions***

The following questions pertain to video [*state the video number that was just viewed*].

- In your own words, what was the main message from this video?
- What information discussed in the video was new to you?
- We want to make sure everyone understands what we are trying to say in our videos. Think about other people like yourself who might see this video. Do you remember a word or phrase they might not know that we can explain better?
- What could we change about this video to make it more interesting or more motivating for you?
- This video was designed to be culturally appropriate for [*RA state: Mexican/Central/South American*] smokers with HIV. What can we do to improve the cultural tailoring of the video?

**Alternate wording of the question above:**

- - By cultural tailoring, we are referring to adapting the content of the video to the [*RA state: Mexican/Central/South American*] culture. What can we do to improve this adaptation?

*Check the participant’s quantitative responses for this video. For each item where they responded either “Neither Agree nor Disagree”, “Strongly Disagree,” or “Disagree,” ask:*

- I see that you responded neutral or disagreed with the statement “[*repeat the statement they disagreed with*].” Tell me about your “neutral” or "disagree" response here. I'd like to understand that.

***Final Interview Questions***

That was our final video. Thank you for watching. [*Pull up screen containing screenshot of each video, divided into their groups*].

Here is a screenshot of all the videos you watched just to help you remember them.

Together, the four videos you just watched covered different aspects of the same topic: [*state Video Group name*].

- Out of these 4 videos, which was your favourite?
- What was it about the video that made it your favourite?
- We may not be able to use all of these videos in the final version of the program. If we have to cut one of the 4 videos in this group, which should we cut?
  - Tell me about your recommendation. I’m interested to hear what made that one your selection.
  - Let’s say we could redo that video. What could we do to improve the video that you cut?
- Thinking about all the videos you watched as a whole, would you consider them to be low, average, or high quality compared to the other videos you’ve seen?
- Which video stuck with you the most?
  - What about it stuck with you?
  - Can you give me an example?
- What did you think of the narration?
- What did you think about the animation?
  - Did the animations consistently match the topic of the video?
- How helpful do you think this video would be in helping someone who is trying to improve their health?
- These videos were designed to be culturally appropriate for [*RA state: Mexican/Central/South American*] smokers with HIV. How effective were the videos in capturing the cultural aspect of this group?

**Alternate wording of the question above:**

- We would like to make sure that these videos are appropriate for ____ smokers with HIV. Think back to the videos you watched.
  - Can you think of an example where one of the videos talked about smoking or HIV in a way that fit with ______ culture?
  - Can you think of an example where one of the videos talked about smoking or HIV in a way that would not fit with _____ culture?
- What else would you would like to share about the videos we watched today?

Finally, thinking about when you decided to participate in this study and completed the online screener for the study…

- What were some reasons you wanted to participate?
- What might be some reasons other people would not want to participate in a study like this?
- What could we do to help people feel more comfortable or be more likely to participate in a study like this?

Thank you for taking the time to talk with us about the study. That is the end of our questions for you.
